# Metrics from Wearable Devices as Candidate Predictors of Antibody Response Following Vaccination against COVID-19: Data from the Second TemPredict Study

**DOI:** 10.3390/vaccines10020264

**Published:** 2022-02-09

**Authors:** Ashley E. Mason, Patrick Kasl, Wendy Hartogensis, Joseph L. Natale, Stephan Dilchert, Subhasis Dasgupta, Shweta Purawat, Anoushka Chowdhary, Claudine Anglo, Danou Veasna, Leena S. Pandya, Lindsey M. Fox, Karena Y. Puldon, Jenifer G. Prather, Amarnath Gupta, Ilkay Altintas, Benjamin L. Smarr, Frederick M. Hecht

**Affiliations:** 1 Osher Center for Integrative Health, University of California San Francisco, San Francisco, CA 94115, USA; wendy.hartogensis@ucsf.edu (W.H.); Anoushka.Chowdhary@ucsf.edu (A.C.); Claudine.Anglo@ucsf.edu (C.A.); danouveasna@gmail.com (D.V.); Leena.Pandya@ucsf.edu (L.S.P.); Lindsey.Fox@ucsf.edu (L.M.F.); Karena.puldon@ucsf.edu (K.Y.P.); Jenifer.Prather@ucsf.edu (J.G.P.); rick.hecht@ucsf.edu (F.M.H.); 2 Halıcıoğlu Data Science Institute, University of California San Diego, San Diego, CA 92093, USA; pkasl@ucsd.edu (P.K.); jonatale@ucsd.edu (J.L.N.); a1gupta@ucsd.edu (A.G.); ialtintas@ucsd.edu (I.A.); bsmarr@eng.ucsd.edu (B.L.S.); 3 Department of Management, Zicklin School of Business, Baruch College, The City University of New York, New York, NY 10010, USA; Stephan.dilchert@baruch.cuny.edu; 4 San Diego Supercomputer Center, University of California San Diego, San Diego, CA 92093, USA; sudasgupta@ucsd.edu (S.D.); shpurawat@sdsc.edu (S.P.)

**Keywords:** COVID-19, antibody responses, mRNA vaccines, wearable devices, skin temperature, heart rate, heart rate variability, sleep

## Abstract

There is significant variability in neutralizing antibody responses (which correlate with immune protection) after COVID-19 vaccination, but only limited information is available about predictors of these responses. We investigated whether device-generated summaries of physiological metrics collected by a wearable device correlated with post-vaccination levels of antibodies to the SARS-CoV-2 receptor-binding domain (RBD), the target of neutralizing antibodies generated by existing COVID-19 vaccines. One thousand, one hundred and seventy-nine participants wore an off-the-shelf wearable device (Oura Ring), reported dates of COVID-19 vaccinations, and completed testing for antibodies to the SARS-CoV-2 RBD during the U.S. COVID-19 vaccination rollout. We found that on the night immediately following the second mRNA injection (Moderna-NIAID and Pfizer-BioNTech) increases in dermal temperature deviation and resting heart rate, and decreases in heart rate variability (a measure of sympathetic nervous system activation) and deep sleep were each statistically significantly correlated with greater RBD antibody responses. These associations were stronger in models using metrics adjusted for the pre-vaccination baseline period. Greater temperature deviation emerged as the strongest independent predictor of greater RBD antibody responses in multivariable models. In contrast to data on certain other vaccines, we did not find clear associations between increased sleep surrounding vaccination and antibody responses.

## 1. Introduction

Vaccines for COVID-19 have been remarkably effective in preventing severe disease, with reductions in the risk of severe disease in the 90% range [1,2]. Existing COVID-19 vaccines do not eliminate the risk of severe disease, however [3], and there is also concern that protection may wane over time [4]. The level of protection against COVID-19 has been shown to be strongly correlated with the level of antibodies directed at the SARS-CoV-2 viral spike protein—antibodies that are capable of viral neutralization [5]. The emergence of SARS-CoV-2 variants that have reduced sensitivity to antibodies generated by existing vaccines, such as the Omicron variant, have also increased the importance of high levels of antibodies post-vaccination to achieve good disease protection [6]. This raises two questions: (1) Are there modifiable factors that influence the level of antibodies generated by vaccination? (2) Are there elements of the physiological response to COVID-19 vaccination that are associated with greater antibody responses?

Prior data suggest that sleep duration just before and after vaccination is associated with the level of antibody responses to vaccination. For example, in an experimentally induced sleep restriction model, less sleep in the nights prior to influenza vaccination predicted lower responses to vaccination [7]. Similarly, relative to a control group that had a normal night’s sleep, participants whose sleep was restricted the night after receiving hepatitis A vaccination developed only half the antibody response [8]. In the case of COVID-19 vaccination, this suggests that a longer sleep duration prior to and just after vaccination might increase the likelihood of stronger antibody responses.

COVID-19 vaccinations result in physiological responses that are short in duration but can be quite noticeable, including fever, chills, and fatigue [1,2]. These physiological responses can differ considerably across individuals. The CDC website [9] on COVID-19 vaccination states that these possible side effects “are normal signs that your body is building protection”. This raises the question of whether greater physiological responses following vaccination might indicate that greater immune responses are developing. Limited data, however, have been published addressing this issue. One report did not find a correlation between vaccine side effects and neutralizing antibodies [10]. A second study found that reporting clinically significant side effects after receiving the Moderna-NIAID vaccine was associated with higher median IgG measurements [11].

We sought to shed light on these questions by analyzing data from the second TemPredict study, which took place in the spring of 2021, just as COVID-19 vaccinations were becoming widely available. In this study, we tested whether a wearable device (Oura Ring), which collects data on dermal temperature, heart rate, heart rate variability, and various sleep metrics (total sleep duration, deep sleep duration, and rapid eye movement (REM) sleep duration) could be used to detect the early period of COVID-19 disease. Prior data suggest that wearable devices can detect changes in physiology that follow mRNA vaccination against COVID-19 [12]. Most participants received a COVID-19 vaccine during the study. To assess antibody responses, we measured antibodies to the SARS-CoV-2 receptor binding domain (RBD) [13] at the end of the study period. Antibodies to the SARS-CoV-2 RBD correlate closely with viral neutralization, providing a simple serologic assay that is related to the level of immune protection achieved by vaccination [14]. In the current analysis, we used physiological measures collected by the Oura Ring around the time of COVID-19 vaccination to assess possible predictors of antibody response as indexed by antibodies to the SARS-CoV-2 RBD.

## 2. Materials and Methods

We initiated the second TemPredict study in December of 2020 to assess whether an algorithm derived from physiological metrics collected by an off-the-shelf wearable device (Oura Ring) could be used to detect COVID-19 infection in real-time. An additional aim of this study was to assess whether data from this device could predict antibody response quantified as antibodies to SARS-CoV-2 spike protein receptor binding domain (RBD), which is the focus of the current report.

### 2.1. Study Participants

*Recruitment.* We recruited participants residing in the United States who already possessed Oura Rings by sending them email invitations. These email invitations included a link to an online consent survey. We also recruited participants who worked at participating sites (e.g., teachers, firefighters, and other first responders) by enlisting leadership at these sites to assist in recruitment. We mailed these sites recruitment materials, including study flyers and Oura Ring sizing kits, which contained plastic rings for prospective participants to try on to determine their size. We provided Oura Rings to interested individuals at these sites after they provided their size information to study coordinators.

*Eligibility and Consent*. Eligible participants were at least 18 years of age, possessed a smartphone that could pair with their Oura Ring, resided in the United States, did not previously have COVID-19 infection (verified through laboratory testing during enrollment), and could communicate in English. For this analysis, of the 2055 participants who completed the overall study, we first excluded participants who had a positive SARS-CoV-2 nucleocapsid antibody test at the end of the study, indicating COVID-19 infection during the study period (*n* = 56). We then excluded participants who were not fully vaccinated at least 7 nights prior to their final blood draw (*n* = 715). We then excluded participants who did not have at least 7 nights of physiological data within the timeframe used to develop the pre-vaccination baseline period (night −14 to night −4 prior to first vaccination or who lacked data for at least one night adjacent to vaccination; *n* = 105).

The University of California San Francisco (UCSF) Institutional Review Board (IRB, IRB# 20-30408) and the U.S. Department of Defense (DOD) Human Research Protections Office (HRPO, HRPO# E01877.1a) approved of all study activities, and all research was performed in accordance with relevant guidelines and regulations. All participants provided electronic (written) informed consent, and this research was conducted according to the principles expressed in the Declaration of Helsinki. Participants to whom we provided Oura Rings kept the devices following their participation; we did not otherwise compensate participants for participation.

### 2.2. Measures

*Questionnaires:* Beginning in December 2020, participants completed several online surveys. They first completed a baseline survey that collected demographic and health information. They also completed daily and monthly surveys on which they reported COVID-19 symptoms, COVID-19 diagnosis, and COVID-19 exposures. Within these surveys, participants also reported whether they had been vaccinated against COVID-19, and if so, which vaccine they received (Pfizer-BioNTech, Moderna-NIAID, or Johnson & Johnson-Janssen) as well as their injection dates. After participants reported on vaccine type and dates of vaccine injections, their surveys were customized such that they were not asked these questions in duplicate on future dates.

*Antibody testing*. We tested participants for antibodies to the SARS-CoV-2 nucleocapsid protein (Test# 164068, LabCorp, Inc.) during enrollment (December 2020 through early April 2021) and at the end of their participation (April and May 2021). The SARS-CoV-2 nucleocapsid antibody test becomes positive following COVID-19 infection, but vaccination does not cause individuals to generate antibodies to this part of the virus. Participants were required to have a negative nucleocapsid antibody test at enrollment as we excluded participants with evidence of prior COVID-19 infection. At the end of the study period (late April and May 2021), we also tested participants for antibodies to the SARS-CoV-2 RBD with the LabCorp Semi-Quantitative Total Antibody, Spike assay (Test# 164090, LabCorp, Inc.), which used the Roche Elecsys Anti-SARS-CoV-2 S assay performed on a COBAS e602 module [15]. The specificity and sensitivity (≥14 days post-PCR diagnosis of COVID-19 infection) for the Elecsys Anti-SARS-CoV-2 S immunoassay is reported to be 99.95% (95% CI: 99.87–99.99) and 97.92% (95% CI: 95.21–99.32), respectively [16]. The dynamic range reported for this assay during the time when most of the study assays were performed was from 0.4 IU/mL to 2500 IU/mL, with a clinical cut-off value for positive results of 0.8 U/mL. Prior to 3 May 2021, LabCorp reported results using an upper detection limit of 250 IU/mL, after which LabCorp changed assay procedures to quantitate antibody levels up to 2500 IU/mL. Fifty-seven participants completed the RBD antibody test before 3 May 2021 and had a result of “>250 IU/mL”.

*Wearable device data (device-generated metrics).* Participants wore the Oura Ring (Generation 2), a commercially available wearable sensor device (Oura Health, Oulu, Finland), on a finger of their choosing. The Oura Ring connects to the Oura App (available from the Google Play Store and the Apple App Store) via Bluetooth. Users can wear the ring continuously in both wet and dry environments.

The Oura Ring generates physiological metrics by aggregating data gathered from on-device sensors. These high-resolution metrics are transformed into summary metrics before transmission to a smartphone app. These device-generated metrics include nightly summary variables of dermal temperature deviations, resting heart rate (HR), resting heart rate variability (HRV), and respiratory rate (RR). The Oura Ring Gen2 assesses HR, HRV, and RR from a photoplethysmogram (PPG) signal generated at 250 Hz. The Oura ring calculates HR, HRV, and RR from inter-beat intervals (IBI), which the Oura Ring only generates during periods of sleep. The Oura Ring calculates HRV in the form of the root mean square of the successive differences (RMSSD). Tri-axial accelerometers estimate activity metrics as metabolic equivalents (MET) reported at 10–60 Hz during both sleep and wake periods, and sleep stages at 5 min resolution. The Oura Ring assesses temperature by using a negative temperature coefficient (NTC) thermistor (resolution of 0.07 °C) on the internal surface of the ring. The sensor registers dermal temperature readings from the palm side of the finger base every 60 s. The temperature deviation metric is computed as the difference between a user’s average overnight temperature and their longer-term baseline, calculated using a rolling window roughly equal to the prior two months. The Oura Ring also outputs sleep metrics that include minutes of light sleep (non-rapid-eye movement [NREM] stages 1 and 2), deep sleep (NREM stages 3 and 4), rapid eye movement (REM) sleep, and total sleep time. We examined these metrics (temperature deviation, HR, HRV, RR, REM sleep duration, deep sleep duration, and total sleep duration) in the present analyses.

*Vaccination.* Participants reported the dates on which they received injections of one of the three vaccines available in the United States (Pfizer-BioNTech, Moderna-NIAID, or Johnson & Johnson-Janssen) between December 2020 and May 2021.

### 2.3. Analytic Plan

*Outcome.* For correlation analyses, we treated values of “>2500 IU/mL” as 2500 IU/mL (*n* = 474). We omitted participants who received a value of “>250 IU/mL;” *n* = 51) from primary analyses. However, we reported analyses including these values as 250 in supplementary results.

*Predictors.* We used device-generated values of each metric two nights before and three nights after each injection (nights −2, −1, 0, 1, 2, where 0 represents the night of the day when vaccination occurred). We established a pre-vaccination baseline period for each participant from 14 nights to 4 nights prior to the first vaccine injection (night −14 to night −4). We calculated values of each device-generated metric adjusting for this pre-vaccination baseline period by converting each physiological metric to a z-score using participants’ respective individual means and standard deviations from the pre-vaccination baseline period (this transformation allows analysis of relative change, without the need for individual device calibration). We examined device-generated metrics from all nights surrounding each injection (−2, −1, 0, 1, 2) and device-generated metrics adjusted for the pre-vaccination baseline period as predictors of RBD antibody responses. Notably, all device-generated metrics reflect values solely from the prior night, except the temperature deviation metric (computed as the difference between the prior night’s value as a deviation from an average derived of the prior two months). The temperature deviation metric adjusted for the pre-vaccination baseline period, therefore, reflects a difference in two deviation metrics: the difference between a participant’s (1) deviation on a particular night surrounding injection and (2) average deviation during the pre-vaccination baseline period.

If a participant was missing device-generated metrics from a particular night, we did not include them in analyses for that night. We excluded participants who had a positive SARS-CoV-2 nucleocapsid antibody test at the end of the study, who did not receive their second injection (Moderna-NIAID and Pfizer-BioNTech) at least 7 nights prior to their final blood draw, who did not have at least 7 nights of physiological data within pre-vaccination baseline period (night −14 to night −4), and who had a threshold value of “>250 IU/mL” on the outcome.

*Statistical analyses.* We conducted analyses separately for each type of vaccine (Johnson & Johnson-Janssen, Moderna-NIAID, Pfizer-BioNTech). We also analyzed the data from both mRNA vaccines combined (Moderna-NIAID and Pfizer-BioNTech). First, we conducted Spearman rank-order correlations between RBD antibody responses and device-generated metrics, before and after adjusting for the pre-vaccination baseline period. We also examined correlations between metrics assessed during the pre-vaccination baseline period and RBD antibody responses. We repeated these analyses retaining participants (*n* = 51) with RBD values of “>250 IU/mL” as “250 IU/mL” and include these results in supplementary tables (Supplementary Materials Tables S1–S3). We replicated these analyses using Kendall rank-order correlation analyses (Kendall’s tau –b) [17] because this approach directly accounts for tied ranks and manages Type I error rates better [18,19,20], and include these results in supplementary tables (Supplementary Materials Tables S4–S6). We used these two approaches to evaluate whether these ordinal correlation analyses would yield a similar pattern of results.

Second, we used the results of the bivariate correlational analyses to inform variable selection for multivariate regression models that assessed which device-generated metrics independently predicted RBD antibody responses. Based on results from the Spearman correlations, we combined data from the mRNA vaccine recipients (Pfizer-BioNTech and Moderna-NIAID) from night 0 after the second injection to predict RBD antibody responses from device-generated metrics before and after adjusting for the pre-vaccination baseline period. We included device-generated metrics that had associations in Spearman correlation analyses (in the combined mRNA sample) before and/or after adjusting for the pre-vaccination baseline period with the RBD antibody responses with *p*-values < 0.1. Due to the proportion of observations with right-censored values of the outcome variable, we adopted a semi-parametric approach using Cox regression models [21], using the RBD antibody result as the dependent variable. Because these analyses used Cox regression to assess differences in antibody levels (rather than time to event typically used in Cox regression), we reported coefficients rather than hazard ratios usually reported with Cox analyses. Coefficients offer insight into the direction and magnitude of associations between the RBD antibody responses and device-generated metrics [22]. We found neither strong nor linear effects of time on antibody titer during the study period. As a result, we did not include temporal parameters in the Cox regression models.

## 3. Results

**Figure 1:**
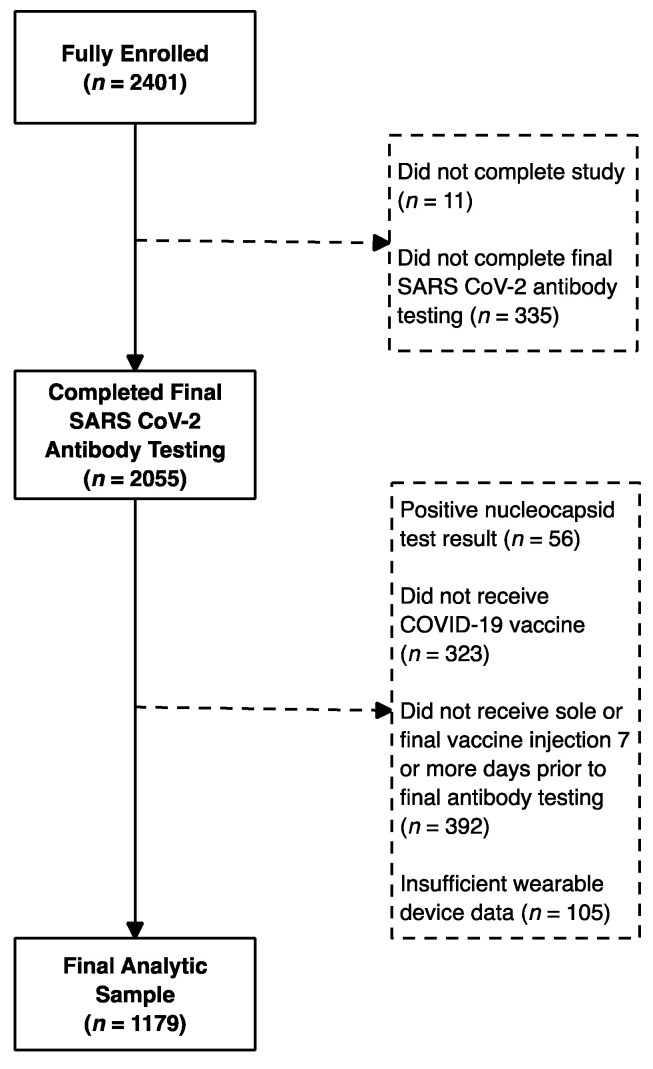
Participant flow through the study.

We enrolled 2392 participants in the second TemPredict study (Figure 1 and Table 1). After excluding participants who did not receive their second injection (Moderna-NIAID and Pfizer-BioNTech) at least 7 nights prior to their final blood draw, who did not have at least 7 nights of physiological data within the pre-vaccination baseline period (night −14 to night −4), who had a value of “>250 IU/mL” on the RBD antibody assay, or who had a positive value on their second nucleocapsid antibody test, there were 1179 participants eligible for this analysis. Of these participants, 107 received Johnson & Johnson-Janssen COVID-19 vaccine, 366 received the Moderna-NIAID vaccine, and 706 received the Pfizer-BioNTech vaccine (Table 1). Of the 1179 participants included in this analysis, 474 had a result of “>2500 IU/mL” on the RBD antibody test, indicating substantial right censoring (40.2%) of the data. Four participants in the analysis dataset had a left-censored RBD value (“<0.4 IU/mL”). Participants in the analytic sample obtained the RBD test an average of 38 days (SD: 30 days) after the final vaccine injection. By vaccine types, the mean (SD) days from final vaccination to RBD testing were: Moderna-NIAID 35 (24), Pfizer-BioNTech 40 (34), and Johnson & Johnson-Janssen 39 (14).

### 3.1. Spearman Correlations

**Figure 2:**
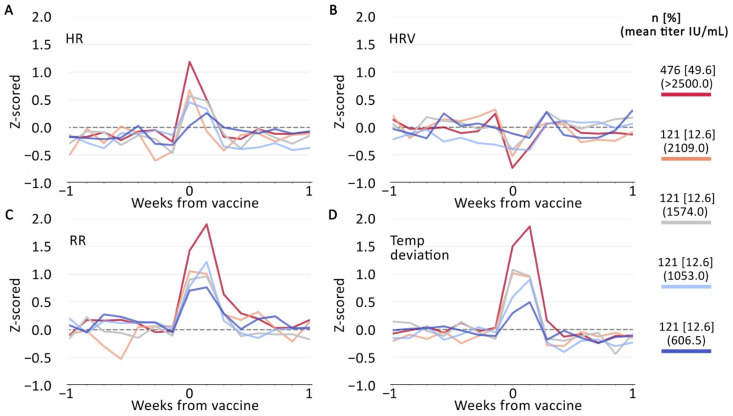
Plots depicting (**A**) changes in heart rate (HR); (**B**) heart rate variability (HRV); (**C**) respiratory rate (RR); and (**D**) temperature deviation the nights surrounding the second injection for Pfizer-BioNTech and Moderna-NAIAD vaccine recipients, combined, Values are z-scored for participants’ pre-vaccination baseline period (see Materials and Methods).

Changes in device-generated metrics during night 0 (the night immediately following the second injection) tended to show a stronger pattern of associations with RBD antibody responses than these metrics the night immediately following the first injection (Table 2 and Figure 2). In some cases, these associations were also evident the following night (night 1) after the second injection.

#### 3.1.1. Device-Generated Metrics

Greater HR and temp deviation on night 0 after the second injection for both the Moderna-NIAID (HR: rho = 0.138, *p* = 0.012; temp deviation: rho = 0.123, *p* = 0.026) and Pfizer-BioNTech (HR: rho = 0.176, *p* < 0.001; temp deviation: rho = 0.152, *p* < 0.001) were associated with greater RBD antibody responses (Table 2). Additionally, on night 0 after the second injection, lower HRV values were associated with greater RBD antibody responses for Pfizer-BioNTech (HRV: rho = −0.092, *p* = 0.022), and these associations were in the same direction, but not statistically significant, for Moderna-NIAID (HRV: rho = −0.056, *p* = 0.312). The associations between RBD antibody responses and HR (Pfizer-BioNTech only) and temp deviation (Pfizer-BioNTech and Moderna-NAIAD) were also evident the following night (night 1) after the second injection. Correlations between device-generated metrics and antibody responses were in the same direction and often had similar rho values for participants who received the Johnson & Johnson-Janssen vaccine, but none of these correlations were statistically significant in the smaller group that received this vaccine.

Analyzing the two mRNA vaccines in combination (Moderna-NIAID and Pfizer-BioNTech) yielded similar associations between device-generated metrics and RBD antibody responses (Table 3). HR, temp deviation, and HRV from night 0 after the second injection were significantly associated with RBD antibody responses (HR: rho = 0.197, *p* < 0.001; temp deviation: rho = 0.238, *p* < 0.001; HRV: rho = −0.118, *p* < 0.001). In analyses combining participants who received either of the two mRNA vaccines, there was a statistically significant inverse correlation between deep sleep on night 0 after the second injection and RBD antibody responses (Deep: rho = −0.079, *p* = 0.014). The associations between RBD antibody responses and HR, RR, and temp deviation were also evident the following night (night 1) after the second injection.

Replicating these analyses retaining participants whose RBD antibody responses were “>250 IU/mL” as 250 IU/mL showed similar patterns of results (Supplementary Materials Tables S1 and S2). Repeating these analyses using Kendall rank-order correlation coefficients demonstrated similar patterns of results (Supplementary Materials Tables S4 and S5).

#### 3.1.2. Device-Generated Metrics Adjusted for Pre-Vaccination Baseline Period

Adjusting for the pre-vaccination baseline period strengthened associations between device-generated metrics and RBD antibody responses revealed significant associations between RBD antibody values and additional metrics (Table 4). Specifically, greater increases in HR and temp deviation on night 0 after the second injection adjusted for the pre-vaccination baseline period for both Moderna-NIAID (HR: rho = 0.148, *p* = 0.007; temp deviation: rho = 0.158, *p* = 0.004) and Pfizer-BioNTech (HR: rho = 0.124, *p* = 0.002; temp deviation: rho = 0.152, *p* < 0.001) were associated with greater RBD antibody responses. Additionally, on night 0 after the second injection, larger decreases in HRV and deep sleep, and a larger increase in RR, were associated with greater RBD antibody responses for Moderna-NIAID (HRV: rho = −0.119, *p* = 0.031; RR: rho = 0.139, *p* = 0.012) and Pfizer-BioNTech (HRV: rho = −0.182, *p* < *0*.001; RR: rho = 0.114, *p* = 0.004). Pfizer-BioNTech also demonstrated an additional association between larger decreases in deep sleep and greater RBD antibody responses (Deep: rho = −0.120, *p* = 0.003). The associations between RBD antibody responses and both RR and temp deviation for each Pfizer-BioNTech and Moderna-NAIAD were also evident the following night (night 1) after the second injection. We did not observe these patterns for Johnson & Johnson-Janssen.

When we combined participants who received either of the two mRNA vaccines (Moderna-NIAID and Pfizer-BioNTech), the associations between device-generated metrics adjusted for pre-vaccination baseline and RBD antibody responses demonstrated greater statistical significance (Table 3). HR, HRV, RR, temp deviation, and deep sleep from night 0 after the second injection were significantly associated with RBD antibody responses. The associations between RBD antibody responses and HRV, HR, RR, and temp deviation were also statistically significant the following night (night 1) after the second injection. Additionally, greater HRV the night prior to the second injection (night −1) was significantly associated with greater RBD antibody responses (rho = 0.07, *p* = 0.024).

Replicating these analyses retaining participants whose RBD antibody responses were “>250 IU/mL” as 250 IU/mL showed similar patterns of results (Supplementary Materials Tables S2 and S3). Repeating these analyses using Kendall rank-order correlation coefficients demonstrated similar patterns of results (Supplementary Materials Tables S5 and S6).

#### 3.1.3. Device-Generated Metrics during the Pre-Vaccination Baseline Period

Among participants who received the Johnson & Johnson-Janssen vaccine, we did not observe any statistically significant (*p* < 0.05) correlations using Spearman rank order or Kendall rank correlation coefficients between baseline values of sleep duration, REM sleep, deep sleep, HRV, HR, RR, or temperature deviation and antibody responses. Among participants who received the Moderna-NIAID vaccine, we observed a correlation between temperature deviation and antibody response such that lower temperature deviation was associated with greater antibody response (Spearman rank order: rho = −0.108, *p* = 0.044; Kendall Tau: **τ** = −0.081, *p* = 0.049). Among participants who received the Pfizer-BioNTech vaccine, we observed a correlation between respiration rate and antibody response such that lower respiration rate was associated with greater antibody response (Spearman rank order: rho = −0.092, *p* = 0.017; Kendall Tau: **τ** = −0.064, *p* = 0.016).

### 3.2. Multivariate Models

Based on the results of bivariate analyses, we focused multivariate analysis on night 0 after the second vaccine injection, using the combined mRNA vaccine (Pfizer-BioNTech and Moderna-NIAID) participants. In Cox regression models, temp deviation on night 0 after the second injection was a statistically significant predictor of RBD antibody responses in models before and after adjusting for the pre-vaccination baseline period (Table 5). In the model unadjusted for the pre-vaccination baseline period, greater HR on night 0 after the second injection was also a statistically significant predictor of greater RBD antibody responses.

## 4. Discussion

We found that physiological metrics from an off-the-shelf wearable device on the two nights following the second dose of an mRNA-based COVID-19 vaccine were associated with RBD antibody responses. Using the device-generated metrics adjusted for the pre-vaccination baseline period, we found that both increased temperature deviation and heart rate (HR) and decreased heart rate variability (HRV) the night immediately following the second mRNA vaccine injection correlated with higher RBD antibody responses. In bivariate analyses using a standardized difference from the pre-vaccination baseline period for each physiological metric, we found that increased HR, temperature deviation, and RR, as well as decreased HRV and deep sleep, were each associated with higher RBD antibody responses for individuals who received the mRNA vaccines. We did not, however, find a meaningful pattern of associations between participants’ device-generated metrics during the pre-vaccination baseline period and antibody responses. Although one earlier study did not find an association between vaccine-related side effects and antibody levels [10], a second study did report higher antibody levels in individuals with clinically significant side effects [11]. Neither study, however, reported actual antibody levels in relation to side effects. Importantly, rather than relying on participant-reported side effects, our study assessed objective (continuously assessed) physiological measures as predictors of vaccine responses. Data presented here speak to recent calls for examining wearable device data in tandem with immune responses to vaccines [12]. The continuous predictors in these analyses may have been more sensitive to the effects of vaccination in generating systemic inflammatory responses.

In a multivariate model predicting RBD antibody responses from device-generated metrics, we found that increased HR and temperature deviation independently predicted greater RBD antibody values for individuals who received the mRNA vaccines. In an identical model adjusting for the pre-vaccination baseline period, we found that dermal temperature was the sole statistically significant independent predictor of greater RBD antibody values in this same sample. Prior research has focused on identifying the roles of behavioral and psychological factors, in particular, sleep parameters, in vaccine responses. Short sleep duration prior to vaccination against influenza reduces antibody responses in both observational and experimental sleep deprivation models [7,23]. These observations have driven hypotheses that a longer sleep duration prior to vaccination against COVID-19 might boost host immune responses [24]. Ongoing research is studying the effects of shift work and short sleep on antibody response following mRNA-based COVID-19 vaccination [25]. Our data did not demonstrate associations between pre-vaccination sleep duration and antibody response among individuals receiving mRNA-based COVID-19 vaccines. In contrast to prior findings with other vaccines, we found that less deep sleep (NREM stages 3/4 sleep) the night immediately *after* receiving an mRNA-based vaccine against COVID-19, both in absolute terms and relative to one’s pre-vaccination levels, was associated with greater antibody responses. Rather than implicating reduced deep sleep after vaccination as a mechanism driving antibody response, a more likely explanation is that individuals experiencing more noticeable discomfort, such as arm pain, fever, chills, or other symptoms that can follow COVID-19 vaccination [9] may have experienced sleep disruptions. Consistent with this explanation, sleep duration was not a significant predictor of vaccine responses in multivariate models. Future research should capture diversified self-reports of the effects of post-vaccination symptomology, including their perceived effects on sleep factors (e.g., duration, restfulness) to further explore this hypothesis.

Mediators of systemic inflammatory responses, such as COX-2, are associated with fever as well as with the development of certain vaccine responses [26]. As antipyretic analgesics, including acetaminophen [27] and non-steroidal anti-inflammatory drugs, can inhibit COX-2, these data have raised concerns that antipyretic analgesics might blunt certain vaccine responses if given at the time of vaccination. Randomized, controlled trial results in children have shown that prophylactic administration of antipyretic drugs at the time of vaccination led to significantly lower antibody responses to multiple vaccines [28]. Consistent with these data, the CDC recommends not taking antipyretic analgesics before the COVID-19 vaccination [29]. It is also possible that the use of antipyretic analgesics following vaccination may blunt immune responses. However, few randomized controlled trials have formally examined this issue [26]. The CDC website suggests that one “talk to a doctor about taking over-the-counter medication, such as ibuprofen, acetaminophen, aspirin (only for people age 18 or older), or antihistamines for any pain and discomfort experienced after getting vaccinated” [9,29]. To date, no trials that we are aware of have examined the role of fever or the impact of antipyretic medications on antibody responses following vaccination with mRNA vaccines. Although our data raise potential concerns that antipyretic analgesics might blunt COVID-19 vaccine responses, as temperature elevation was associated with greater antibody responses, our data did not directly address the effect of these medications and did not shed light on whether antipyretics influence immune pathways involved in the generation of immune responses to COVID-19 vaccines. Taken together, prior research [26] and our data highlighted the potential importance of further research testing the effects of antipyretic medications used after receiving COVID-19 vaccines on antibody responses.

Psychological factors, including psychological stress, can impact immunological responses to vaccination, and clarifying their impact on COVID-19 vaccination may be important for developing interventions to optimize antibody response [30]. For example, prior research demonstrated the negative effects of stress on immune response following vaccination against Hepatitis B [31]. More recent work has shown negative effects of poor sleep prior to vaccination against influenza [24], and subsequent studies have replicated similar patterns across multiple types of vaccinations [30]. Decreased HRV can indicate increased sympathetic nervous system tone. Researchers have thus operationalized psychological stress using measures of heart rate variability (HRV), although the correlation of heart rate variability with stress is imperfect and can depend on several moderators, including contextual factors [32]. We found that in the combined mRNA vaccine group, on the night immediately prior to the second injection adjusted for baseline (night −1), HRV was positively correlated with antibody responses. This suggests that lower HRV (consistent with greater psychological stress [32,33]) was associated with lower antibody responses. This association changed direction in the following night (night 0) such that HRV was negatively correlated with antibody responses. This likely represents the effect of increased systemic inflammatory responses to vaccination, resulting in elevated temperature and HR, and decreased HRV, which in turn was associated with greater antibody responses. Consistent with this explanation, HRV did not significantly predict antibody response in multivariate models with other metrics (i.e., temperature), however, suggesting HRV after vaccination was not independently associated with antibody responses. Future research on this issue should measure stress more broadly (both self-report and physiological metrics of psychological stress) as predictors of antibody response to better clarify the role of stress in COVID-19 vaccine responses.

Our data have several limitations. We did not collect information on antipyretic or other medication use surrounding the time of vaccine injections, and thus cannot directly assess any effects of antipyretics on vaccine responses. We also did not collect detailed self-report information on post-vaccination symptomology, and we did not collect anthropometric information (e.g., height, weight, blood pressure) from participants as this study was completed by mail and internet only. Future research should collect such information, as emerging data suggests that health metrics, such as body mass index, may be associated with antibody responses [34]. The RBD antibody assay we used had an upper limit of dynamic range of 2500 IU/mL. A substantial proportion of participants achieved antibody levels above this range, resulting in right-censored data. To address this, we used Cox regression, a robust approach for analyzing right-censored data (readers may be more familiar with this approach when analyzing time-to-event data). Future research would benefit from an antibody assay with an extended dynamic range. We used a commercially available RBD antibody assay to assess neutralizing antibody responses rather than more precise but more expensive approaches, such as pseudo virus neutralization assays. Like other studies exploring vaccination responses, our results will become more meaningful once there are enough data to form a scientific consensus on an antibody level that indicates adequate immune protection.

## 5. Conclusions

In conclusion, we found that several physiological metrics generated by an off-the-shelf wearable device in the nights following a second mRNA vaccination against COVID-19 were associated with subsequent levels of RBD antibody levels. In multivariate analyses, we found that elevated temperature was the strongest independent predictor of antibody responses. These findings suggest that off-the-shelf wearable devices collect data that could be useful in predicting immune responses to COVID-19 vaccination, though the clinical implications remain uncertain. Our data suggest that further investigation of the effects of antipyretics on COVID-19 vaccine responses is warranted.

## 6. Patents

Patent application US App. No. 63/287,914 was filed on December 9 by the University of California San Francisco. The application covers use of wearable device data to predict the development of antibodies against COVID-19 following vaccination against COVID-19. A.E.M. and B.L.S. are listed as co-inventors on these applications.

## Figures and Tables

**Table 1:** Participant characteristics.

	Pfizer-BioNTech	Moderna-NIAID	Johnson & Johnson-Janssen	Overall
N	706	366	107	1179
Age (M, SD)	50.4 (11.4)	52.8 (12.0)	49.5 (9.9)	51.0 (11.5)
Biological sex (N, %) Male Female Intersex	334 (47.3) 371 (52.5) 1 (0.1)	166 (45.4) 200 (54.6) 0 (0)	53 (49.5) 54 (50.5) 0 (0)	553 (46.9) 625 (53.0) 1 (0.1)
Race (N, %) African African American Caribbean Caucasian/White East Asian Middle Eastern Native American/Native Alaskan Native Hawaiian/Other Pacific Islander South AsianMore than one race Prefer not to answer/Unknown	1 (0.1) 9 (1.3) 1 (0.1) 603 (85.4) 37 (5.2) 5 (0.7) 3 (0.4) 0 (0) 16 (2.3) 24 (3.4) 7 (1.0)	1 (0.3) 6 (1.6) 1 (0.3) 325 (88.8) 9 (2.5) 3 (0.8) 0 (0) 0 (0) 3 (0.8) 15 (4.1) 3 (0.8)	0 (0) 2 (1.9) 0 (0) 95 (88.8) 4 (3.7) 1 (0.9) 0 (0) 1 (0.9) 1 (0.9) 2 (1.9) 1 (0.9)	2 (0.2) 17 (1.4) 2 (0.2) 1023 (86.8) 50 (4.2) 9 (0.8) 3 (0.3) 1 (0.1) 20 (1.7) 41 (3.5) 11 (0.9)
Hispanic/Latinx (N, %) Yes No Don’t Know/Not Sure Prefer not to answer	30 (4.2) 671 (95.0) 4 (0.6) 1 (0.1)	17 (4.6) 347 (94.8) 1 (0.3) 1 (0.3)	7 (6.5) 100 (93.5) 0 (0) 0 (0)	54 (4.6) 1118 (94.8) 5 (0.4) 2 (0.2)
Education (N, %) Less than a high school diploma High school diploma or GED Some college Associate Degree (e.g., AA, AS) Bachelor’s Degree (e.g., BA, BS) Master’s Degree (e.g., MA, MS) Advanced Degree (e.g., Ph.D., EdD, MD, JD)	0 (0) 5 (0.7) 40 (5.7) 27 (3.8) 270 (38.2) 223 (31.6) 141 (20.0)	0 (0) 3 (0.8) 27 (7.4) 13 (3.6) 131 (35.8) 119 (32.5) 73 (19.9)	0 (0) 3 (2.8) 10 (9.3) 6 (5.6) 40 (37.4) 38 (35.5) 10 (9.3)	0 (0) 11 (0.9) 77 (6.5) 46 (3.9) 441 (37.4) 380 (32.2) 224 (19.0)
RBD Value (N, %) Value of 0 to 2499 IU/mL Value of “>250 IU/mL” Value of “>2500 IU/mL” RBD Value (Median, IQR) Value of 0 to 2499 IU/mL Primary analysis data *	458 (64.9) 33 (4.7) 215 (30.5) 1613.0 [778.4, 2500.0]	89 (24.3) 18 (4.9) 259 (70.8) 2500.0 [2477.0, 2500.0]	107 (100.0) 0 (0) 0 (0) 19.1 [6.1,50.1]	654 (55.5) 51 (4.3) 474 (40.2) 1956.5 [753.3, 2500.0]

Note: IQR = Interquartile Range. * Primary analysis data include values of “>2500 IU/mL” as 2500 IU/mL, excluding values of “>250 IU/mL”.

**Table 2:** Spearman rank-order correlations between RBD antibody responses and device-generated metrics on nights before and after each vaccine injection.

		Metric	Injection 1		Injection 1				Injection 2			
			J&J		Moderna		Pfizer		Moderna		Pfizer	
			rho	*p*	rho	*p*	rho	*p*	rho	*p*	rho	*p*
**Night Relative to Injection**	−2	Sleep Duration−	−0.127	0.207	−0.031	0.571	0.018	0.649	0.069	0.218	−0.023	0.564
		REM Sleep	0.026	0.799	−0.036	0.518	**0.104**	**0.009**	0.008	0.886	0.061	0.126
		Deep Sleep	−0.139	0.166	−0.024	0.657	0.010	0.808	0.070	0.214	0.049	0.219
		HRV (RMSSD)	−0.119	0.236	0.013	0.812	−0.046	0.251	0.007	0.908	0.025	0.536
		HR	−0.038	0.706	0.033	0.547	**0.138**	**0.000**	0.031	0.583	0.063	0.117
		RR	0.072	0.477	−0.048	0.381	−0.067	0.092	**−0.130**	**0.020**	−0.045	0.260
		Temp Deviation	−0.028	0.784	−0.012	0.830	0.052	0.193	0.043	0.439	−0.005	0.891
	−1	Sleep Duration	0.036	0.723	−0.022	0.683	−0.044	0.270	−0.059	0.289	−0.068	0.088
		REM Sleep	0.018	0.856	−0.062	0.255	0.033	0.403	−0.086	0.126	0.001	0.976
		Deep Sleep	0.012	0.908	−0.048	0.375	0.047	0.236	0.005	0.933	0.048	0.235
		HRV (RMSSD)	−0.081	0.421	0.049	0.366	0.001	0.974	0.028	0.614	0.044	0.275
		HR	0.056	0.574	0.021	0.704	**0.103**	**0.009**	0.020	0.726	**0.093**	**0.020**
		RR	0.122	0.223	−0.047	0.387	**−0.094**	**0.018**	−0.048	0.391	**−0.079**	**0.048**
		Temp Deviation	−0.047	0.640	−0.023	0.670	0.002	0.951	0.053	0.342	−0.023	0.568
	0	Sleep Duration	0.125	0.211	0.066	0.235	**−0.080**	**0.044**	−0.003	0.960	−0.033	0.408
		REM Sleep	0.054	0.593	0.045	0.413	−0.002	0.968	0.024	0.659	0.033	0.407
		Deep Sleep	−0.162	0.106	−0.010	0.860	0.005	0.899	−0.025	0.654	−0.058	0.148
		HRV (RMSSD)	−0.047	0.638	0.011	0.838	0.006	0.873	−0.056	0.312	**−0.092**	**0.022**
		HR	0.125	0.213	0.008	0.885	**0.101**	**0.010**	**0.138**	**0.012**	**0.176**	**0.000**
		RR	0.123	0.222	−0.053	0.340	−0.058	0.141	−0.021	0.711	−0.004	0.925
		Temp Deviation	0.155	0.122	−0.009	0.870	−0.003	0.935	**0.123**	**0.026**	**0.152**	**0.000**
	1	Sleep Duration	**−0.202**	**0.044**	−0.053	0.334	−0.002	0.957	0.069	0.221	−0.009	0.823
		REM Sleep	−0.056	0.580	0.023	0.674	0.045	0.258	0.068	0.231	**0.082**	**0.041**
		Deep Sleep	−0.149	0.138	−0.006	0.912	0.042	0.290	−0.090	0.110	0.026	0.522
		HRV (RMSSD)	−0.073	0.471	0.003	0.962	−0.012	0.765	0.011	0.842	−0.016	0.696
		HR	0.049	0.631	0.002	0.968	**0.087**	**0.028**	0.053	0.352	**0.116**	**0.004**
		RR	0.157	0.119	−0.030	0.585	−0.066	0.098	−0.007	0.899	0.049	0.229
		Temp Deviation	0.093	0.356	0.055	0.316	−0.030	0.451	**0.136**	**0.016**	**0.186**	**0.000**
	2	Sleep Duration	0.046	0.646	−0.019	0.735	−0.016	0.684	−0.061	0.282	**−0.106**	**0.008**
		REM Sleep	0.019	0.853	−0.026	0.641	0.032	0.425	−0.038	0.497	−0.001	0.974
		Deep Sleep	0.026	0.795	−0.039	0.478	−0.006	0.870	0.012	0.832	−0.013	0.739
		HRV (RMSSD)	−0.074	0.465	0.038	0.491	0.005	0.891	0.004	0.942	−0.041	0.312
		HR	0.108	0.282	0.033	0.552	0.089	**0.025**	0.041	0.467	**0.100**	**0.013**
		RR	0.144	0.152	0.000	0.997	−0.090	**0.023**	−0.048	0.394	−0.027	0.496
		Temp Deviation	0.187	0.062	0.001	0.987	−0.019	0.641	0.081	0.153	0.021	0.594

Note: See Materials and Methods for variable descriptions. rho = Spearman’s Rank Order Correlation Coefficient; Night relative to injection = −2 as 2 nights before vaccine, −1 as one night before injection, 0 as night immediately after injection, 1 as night the day after injection, 2 as 2 nights after injection; Temp Deviation = Temperature deviation; REM = Rapid Eye Movement; Deep = Stages Non-REM 3 and 4 sleep; HRV = Heart Rate Variability; RMSSD = Root Mean Square of Successive Differences; HR = Heart Rate; RR = Respiratory Rate.

**Table 3:** Spearman rank order correlations between RBD antibody responses and device-generated metrics before and after adjusting for the pre-vaccination baseline period on nights before and after injections for Moderna-NIAID and Pfizer-BioNTech vaccine recipients, combined.

		Metric	Device-Generated Metric				Adjusted for Baseline Period			
			Injection 1		Injection 2		Injection 1		Injection 2	
			rho	*p*	rho	*p*	rho	*p*	rho	*p*
**Night Relative to Injection**	−2	Sleep Duration	−0.001	0.982	0.021	0.519	0.001	0.988	0.062	0.057
		REM Sleep	0.056	0.079	0.059	0.071	0.028	0.380	0.038	0.245
		Deep Sleep	−0.008	0.808	0.042	0.194	−0.004	0.901	0.040	0.221
		HRV (RMSSD)	−0.031	0.338	−0.007	0.819	**−0.065**	**0.043**	0.004	0.904
		HR	**0.100**	**0.002**	0.051	0.120	**0.081**	**0.012**	−0.022	0.496
		RR	−0.041	0.197	**−0.071**	**0.030**	0.058	0.071	−0.011	0.735
		Temp Deviation	0.017	0.603	0.001	0.967	0.044	0.167	0.023	0.488
	−1	Sleep Duration	−0.011	0.729	−0.042	0.199	−0.010	0.764	−0.023	0.488
		REM Sleep	0.036	0.264	−0.015	0.648	0.008	0.798	−0.050	0.123
		Deep Sleep	0.017	0.594	0.031	0.340	0.056	0.079	0.024	0.463
		HRV (RMSSD)	−0.001	0.981	0.043	0.186	0.011	0.741	**0.074**	**0.024**
		HR	**0.070**	**0.029**	0.052	0.114	−0.009	0.773	−0.011	0.746
		RR	−0.056	0.080	**−0.064**	**0.048**	0.009	0.770	−0.026	0.430
		Temp Deviation	0.008	0.813	−0.015	0.644	0.036	0.264	0.018	0.591
	0	Sleep Duration	−0.023	0.476	−0.012	0.704	−0.018	0.565	0.024	0.462
		REM Sleep	0.010	0.755	0.031	0.343	−0.024	0.460	0.022	0.497
		Deep Sleep	−0.025	0.430	**−0.079**	**0.014**	−0.014	0.667	**−0.126**	**0.000**
		HRV (RMSSD)	−0.010	0.749	**−0.118**	**0.000**	−0.007	0.816	**−0.199**	**0.000**
		HR	**0.070**	**0.029**	**0.197**	**0.000**	0.013	0.695	**0.208**	**0.000**
		RR	−0.039	0.221	0.038	0.241	**0.069**	**0.030**	**0.177**	**0.000**
		Temp Deviation	0.006	0.850	**0.238**	**0.000**	0.023	0.471	**0.244**	**0.000**
	1	Sleep Duration	−0.018	0.573	**0.065**	**0.047**	−0.016	0.625	**0.125**	**0.000**
		REM Sleep	0.034	0.289	**0.081**	**0.014**	0.010	0.757	**0.084**	**0.011**
		Deep Sleep	0.014	0.655	−0.012	0.710	0.028	0.381	−0.021	0.522
		HRV (RMSSD)	−0.032	0.317	−0.029	0.385	**−0.080**	**0.013**	**−0.074**	**0.024**
		HR	**0.072**	**0.024**	**0.100**	**0.002**	0.035	0.281	**0.083**	**0.012**
		RR	−0.015	0.643	**0.067**	**0.042**	**0.097**	**0.002**	**0.260**	**0.000**
		Temp Deviation	0.040	0.215	**0.241**	**0.000**	**0.065**	**0.042**	**0.250**	**0.000**
	2	Sleep Duration	0.012	0.714	**−0.084**	**0.010**	0.031	0.329	**−0.083**	**0.011**
		REM Sleep	0.037	0.252	−0.013	0.690	0.031	0.327	−0.048	0.146
		Deep Sleep	−0.024	0.462	−0.020	0.533	−0.018	0.584	−0.031	0.350
		HRV (RMSSD)	−0.001	0.980	−0.031	0.337	0.008	0.799	−0.051	0.120
		HR	**0.068**	**0.034**	**0.068**	**0.038**	0.024	0.457	0.005	0.871
		RR	−0.051	0.111	−0.008	0.816	0.054	0.092	**0.117**	**0.000**
		Temp Deviation	0.013	0.693	**0.068**	**0.039**	0.021	0.507	**0.076**	**0.020**

Note: See Table 2 note. Pre-vaccination baseline period taken from nights −14 to −4 prior to first injection, see Materials and Methods.

**Table 4:** Spearman rank order correlations between RBD antibody responses and device-generated metrics on nights before and after vaccine injections, adjusted for the pre-vaccination baseline period.

		Metric	Injection 1		Injection 1				Injection 2			
			J&J		Moderna		Pfizer		Moderna		Pfizer	
			rho	*p*	rho	*p*	rho	*p*	rho	*p*	rho	*p*
**Night Relative to Injection**	−2	Sleep Duration	−0.095	0.343	−0.029	0.602	0.052	0.194	**0.127**	**0.023**	0.046	0.253
		REM Sleep	−0.052	0.605	−0.051	0.356	**0.080**	**0.043**	0.014	0.800	0.047	0.244
		Deep Sleep	−0.137	0.173	−0.011	0.841	−0.008	0.847	0.073	0.194	0.028	0.478
		HRV (RMSSD)	−0.105	0.297	−0.035	0.526	**−0.101**	**0.011**	0.049	0.384	0.022	0.579
		HR	−0.111	0.268	0.028	0.607	**0.100**	**0.012**	−0.028	0.624	−0.057	0.155
		RR	−0.076	0.450	−0.007	0.906	0.064	0.104	−0.047	0.402	0.037	0.357
		Temp Deviation	−0.052	0.605	0.039	0.479	0.060	0.133	0.076	0.178	−0.005	0.893
	−1	Sleep Duration	0.033	0.740	−0.008	0.878	−0.033	0.408	−0.015	0.793	−0.020	0.613
		REM Sleep	−0.016	0.874	−0.062	0.257	−0.014	0.734	−0.109	0.051	−0.040	0.316
		Deep Sleep	0.062	0.539	−0.029	0.594	0.070	0.079	0.011	0.847	0.018	0.659
		HRV (RMSSD)	−0.077	0.441	0.035	0.522	0.004	0.920	0.054	0.339	0.049	0.220
		HR	0.128	0.199	0.010	0.853	−0.012	0.761	−0.052	0.350	0.023	0.571
		RR	0.007	0.947	−0.007	0.902	0.009	0.816	0.055	0.325	0.004	0.914
		Temp Deviation	0.004	0.971	0.031	0.567	0.003	0.944	0.072	0.201	−0.017	0.678
	0	Sleep Duration	0.166	0.098	0.097	0.079	−0.050	0.207	0.015	0.784	0.022	0.589
		REM Sleep	0.106	0.293	0.051	0.355	−0.044	0.269	0.037	0.501	0.012	0.763
		Deep Sleep	−0.173	0.083	−0.010	0.863	−0.009	0.816	−0.054	0.326	**−0.120**	**0.003**
		HRV (RMSSD)	−0.001	0.993	−0.045	0.422	0.012	0.758	**−0.119**	**0.031**	**−0.182**	**0.000**
		HR	0.125	0.212	−0.026	0.636	−0.018	0.642	**0.148**	**0.007**	**0.124**	**0.002**
		RR	0.113	0.262	0.008	0.881	0.074	0.061	**0.139**	**0.012**	**0.114**	**0.004**
		Temp Deviation	0.141	0.160	0.021	0.710	0.000	0.994	**0.158**	**0.004**	**0.152**	**0.000**
	1	Sleep Duration	**−0.224**	**0.025**	−0.068	0.215	0.026	0.508	0.088	0.120	**0.081**	**0.045**
		REM Sleep	**−0.281**	**0.005**	0.058	0.292	−0.009	0.818	**0.112**	**0.048**	0.065	0.109
		Deep Sleep	−0.152	0.130	0.024	0.667	0.047	0.238	**−0.123**	**0.030**	0.005	0.892
		HRV (RMSSD)	0.002	0.986	−0.042	0.439	−0.060	0.131	−0.004	0.946	−0.058	0.147
		HR	0.042	0.677	−0.049	0.376	−0.005	0.896	0.042	0.460	0.054	0.182
		RR	0.057	0.573	0.103	0.061	0.041	0.299	**0.168**	**0.003**	**0.259**	**0.000**
		Temp Deviation	0.067	0.511	0.094	0.085	−0.009	0.814	**0.169**	**0.003**	**0.182**	**0.000**
	2	Sleep Duration	0.030	0.769	−0.006	0.912	0.034	0.391	−0.066	0.246	−0.064	0.111
		REM Sleep	−0.125	0.214	−0.018	0.740	0.016	0.685	−0.080	0.155	−0.031	0.446
		Deep Sleep	0.052	0.602	−0.018	0.738	−0.013	0.742	0.046	0.415	−0.065	0.106
		HRV (RMSSD)	−0.039	0.701	0.076	0.164	−0.014	0.717	0.004	0.937	**−0.112**	**0.005**
		HR	0.111	0.269	0.010	0.858	0.021	0.602	0.003	0.963	0.033	0.408
		RR	0.082	0.416	0.099	0.069	0.028	0.477	**0.138**	**0.014**	**0.097**	**0.016**
		Temp Deviation	0.195	0.051	0.044	0.422	−0.023	0.561	**0.124**	**0.027**	0.009	0.821

Note: See Table 2 and Table 3 notes for variable descriptions and preparations.

**Table 5:** Multivariate regression models predicting RBD antibody responses from device-generated metrics before and after adjusting for the pre-vaccination baseline period that demonstrated associations with RBD antibody responses in Spearman correlations from night 0 after the second injection for Moderna-NIAID and Pfizer-BioNTech vaccine recipients, combined.

Device-Generated Metric GFI –log2(p) = 44.95	Coefficient (Beta)	95% CI (LB, UB)	Z	*p*
HRV (RMSSD)	−0.002	(−0.007, 0.004)	−0.584	0.559
RR	0.035	(−0.024, 0.093)	1.161	0.246
HR	−0.019	(−0.031, −0.007)	−3.178	0.002
Deep	0.000	(0.000, 0.000)	1.125	0.260
Temp Deviation	−0.515	(−0.707, −0.322)	−5.247	<0.001
**Device-Generated Metric Adjusted for Baseline period** **GFI –log2(p) = 44.65**				
HRV (RMSSD)	0.047	(−0.023, 0.117)	1.308	0.191
RR	−0.042	(−0.091, 0.008)	−1.632	0.103
HR	−0.013	(−0.073, 0.047)	−0.428	0.669
Deep	0.036	(−0.032, 0.104)	1.043	0.297
Temp Deviation	−0.071	(−0.109, −0.034)	−3.748	<0.001

Note: See Table 2 and Table 3 notes for variable descriptions and preparations. In Cox regression models, coefficient values are inverted from the usual linear regression interpretation: Negative coefficients represent decreased hazard, which in this context translates to increased RBD antibody responses. Positive coefficients represent increased hazard, which in this context translates to decreased antibody levels. Because we used antibody titer in place of survival time in these models, coefficients represent the association between the change in the predictor and the rank of the RBD antibody value.

## Data Availability

Oura’s data use policy does not permit us to make the data available to third parties without approval. Therefore, those seeking to reproduce our findings should contact A.E.M. (corresponding author) for an online application to access the study data portal. This application process will require requesters to make a written commitment expressing agreements to not duplicate data, to not share data with third parties, and/or other confidentiality precautions.

## Supplementary Materials

The following supporting information can be downloaded at: https://www.mdpi.com/article/10.3390/vaccines10020264/s1, Table S1: Spearman rank order correlations between RBD antibody responses and device-generated metrics (retaining *n* = 51 values of “>250 IU/mL” as 250 IU/mL) on nights surrounding each injection; Table S2: Spearman rank order correlations between RBD antibody responses and device-generated metrics before and after adjusting for the pre-vaccination baseline period (retaining *n* = 51 values of “>250 IU/mL” as 250 IU/mL) on nights surrounding injections for Moderna-NIAID and Pfizer-BioNTech vaccine recipients, combined; Figure S3: Spearman rank order correlations between RBD antibody responses and device-generated metrics (retaining *n* = 51 values of “>250 IU/mL” as 250 IU/mL) on nights 0, 1, and 2, adjusted for the pre-vaccination baseline period; Table S4: Kendall rank order correlations between RBD antibody responses and device-generated metrics on nights surrounding each injection; Table S5: Kendall rank order correlations between RBD antibody responses and device-generated metrics before and after adjusting by the pre-vaccination baseline period on nights surrounding injections for Moderna-NIAID and Pfizer-BioNTech vaccine recipients, combined; Table S6: Kendall rank order correlations between RBD antibody responses and device-generated metrics on nights 0, 1, and 2, adjusted for the pre-vaccination baseline period.
