# Supplementary material for: Metrics from Wearable Devices as Candidate Predictors of Antibody Response Following Vaccination against COVID-19: Data from the Second TemPredict Study
Source: Vaccines (Basel). 2022 Feb 9;10(2):264. doi: 10.3390/vaccines10020264 (PMC8877860; doi:10.3390/vaccines10020264)
Supplement: Supplementary file 1 [file vaccines-10-00264-s001.zip › vaccines-1547619-supplementary.pdf]

**Table S1**. Spearman rank order correlations between RBD antibody responses and device-generated metrics (retaining *n*=51 values of ">250 IU/ml" as 250 IU/ml) on nights before and after vaccine injections.

| **Night Relative to Injection** | **Metric** | **Injection 1** | | **Injection 1** | | | | **Injection 2** | | | |
|---|---|---|---|---|---|---|---|---|---|---|---|
| | | **J&J** | | **Moderna** | | **Pfizer** | | **Moderna** | | **Pfizer** | |
| | | **rho** | *P* | **rho** | *P* | **rho** | *P* | **rho** | *P* | **rho** | *P* |
| -2 | Sleep Duration | -0.127 | 0.207 | -0.012 | 0.819 | 0.019 | 0.617 | 0.027 | 0.618 | -0.025 | 0.528 |
| | REM Sleep | 0.026 | 0.799 | -0.028 | 0.596 | **0.095** | **0.015** | -0.015 | 0.787 | 0.041 | 0.297 |
| | Deep Sleep | -0.139 | 0.166 | 0.004 | 0.938 | 0.040 | 0.298 | 0.100 | 0.066 | 0.063 | 0.109 |
| | HRV (RMSSD) | -0.119 | 0.236 | 0.039 | 0.467 | -0.053 | 0.172 | 0.010 | 0.861 | 0.010 | 0.800 |
| | HR | -0.038 | 0.706 | 0.028 | 0.595 | **0.149** | **0.000** | 0.022 | 0.684 | **0.085** | **0.030** |
| | RR | 0.072 | 0.477 | -0.073 | 0.170 | -0.037 | 0.336 | **-0.128** | **0.018** | -0.022 | 0.575 |
| | Temp Deviation | -0.028 | 0.784 | -0.064 | 0.234 | 0.061 | 0.115 | 0.011 | 0.841 | -0.010 | 0.789 |
| -1 | Sleep Duration | 0.036 | 0.723 | -0.017 | 0.756 | -0.050 | 0.193 | -0.014 | 0.796 | -0.044 | 0.266 |
| | REM Sleep | 0.018 | 0.856 | -0.058 | 0.278 | 0.012 | 0.753 | -0.055 | 0.314 | 0.004 | 0.915 |
| | Deep Sleep | 0.012 | 0.908 | -0.017 | 0.746 | 0.069 | 0.073 | 0.012 | 0.826 | 0.057 | 0.145 |
| | HRV (RMSSD) | -0.081 | 0.421 | 0.074 | 0.163 | -0.004 | 0.922 | 0.060 | 0.270 | 0.028 | 0.480 |
| | HR | 0.056 | 0.574 | -0.001 | 0.987 | **0.112** | **0.004** | 0.000 | 0.998 | **0.114** | **0.003** |
| | RR | 0.122 | 0.223 | -0.061 | 0.251 | -0.060 | 0.119 | -0.072 | 0.185 | -0.054 | 0.168 |
| | Temp Deviation | -0.047 | 0.640 | -0.046 | 0.390 | -0.017 | 0.654 | 0.054 | 0.325 | -0.009 | 0.820 |
| 0 | Sleep Duration | 0.125 | 0.211 | 0.096 | 0.076 | **-0.082** | **0.034** | 0.001 | 0.991 | -0.027 | 0.482 |
| | REM Sleep | 0.054 | 0.593 | 0.045 | 0.400 | 0.002 | 0.952 | 0.054 | 0.321 | 0.027 | 0.487 |
| | Deep Sleep | -0.162 | 0.106 | -0.006 | 0.913 | 0.025 | 0.520 | -0.022 | 0.680 | -0.029 | 0.452 |
| | HRV (RMSSD) | -0.047 | 0.638 | 0.037 | 0.494 | -0.005 | 0.892 | -0.066 | 0.222 | **-0.090** | **0.020** |
| | HR | 0.125 | 0.213 | 0.012 | 0.829 | **0.104** | **0.007** | **0.148** | **0.006** | **0.185** | **0.000** |
| | RR | 0.123 | 0.222 | -0.059 | 0.275 | -0.034 | 0.380 | 0.000 | 0.996 | 0.025 | 0.531 |
| | Temp Deviation | 0.155 | 0.122 | 0.006 | 0.917 | -0.021 | 0.587 | **0.132** | **0.014** | **0.133** | **0.001** |
| 1 | Sleep Duration | **-0.202** | **0.044** | -0.044 | 0.408 | 0.001 | 0.980 | 0.042 | 0.448 | 0.004 | 0.919 |
| | REM Sleep | -0.056 | 0.580 | 0.028 | 0.600 | 0.045 | 0.252 | 0.045 | 0.417 | **0.082** | **0.038** |
| | Deep Sleep | -0.149 | 0.138 | -0.006 | 0.913 | 0.061 | 0.114 | -0.051 | 0.350 | 0.043 | 0.272 |
| | HRV (RMSSD) | -0.073 | 0.471 | 0.025 | 0.639 | -0.024 | 0.530 | 0.039 | 0.481 | -0.026 | 0.514 |
| | HR | 0.049 | 0.631 | 0.005 | 0.931 | **0.103** | **0.008** | 0.030 | 0.587 | **0.132** | **0.001** |
| | RR | 0.157 | 0.119 | -0.036 | 0.505 | -0.043 | 0.268 | -0.035 | 0.525 | 0.073 | 0.065 |
| | Temp Deviation | 0.093 | 0.356 | 0.045 | 0.400 | -0.040 | 0.302 | 0.096 | 0.081 | **0.172** | **0.000** |
| 2 | Sleep Duration | 0.046 | 0.646 | 0.008 | 0.884 | -0.004 | 0.925 | -0.003 | 0.959 | **-0.091** | **0.020** |
| | REM Sleep | 0.019 | 0.853 | 0.002 | 0.970 | 0.036 | 0.350 | 0.008 | 0.879 | -0.004 | 0.921 |
| | Deep Sleep | 0.026 | 0.795 | -0.018 | 0.732 | 0.006 | 0.878 | 0.044 | 0.424 | -0.002 | 0.955 |
| | HRV (RMSSD) | -0.074 | 0.465 | 0.026 | 0.631 | -0.021 | 0.592 | 0.034 | 0.539 | -0.044 | 0.263 |
| | HR | 0.108 | 0.282 | 0.038 | 0.483 | **0.109** | **0.005** | 0.024 | 0.659 | **0.110** | **0.005** |
| | RR | 0.144 | 0.152 | -0.036 | 0.497 | -0.055 | 0.156 | -0.065 | 0.233 | -0.006 | 0.880 |
| | Temp Deviation | 0.187 | 0.062 | 0.006 | 0.912 | 0.000 | 0.997 | 0.099 | 0.072 | 0.003 | 0.940 |

*Note*. See Table 2 note. Analyses are identical to Table 2, however, retain the *n*=51 participants whose RBD antibody responses were returned from LabCorp as ">250 IU/ml" as having values of 250 IU/ml.

**Table S2**. Spearman rank order correlations between RBD antibody responses and device-generated metrics before and after adjusting for the pre-vaccination baseline period (retaining *n*=51 values of ">250 IU/ml" as 250 IU/ml) on nights surrounding injections for Moderna-NIAID and Pfizer-BioNTech vaccine recipients, combined.

| Night Relative to Injection | | Metric | Device-generated Metric | | | | Adjusted for Baseline period | | | |
|---|---|---|---|---|---|---|---|---|---|---|
| | | | Injection 1 | | Injection 2 | | Injection 1 | | Injection 2 | |
| | | | rho | *P* | rho | *P* | rho | *P* | rho | *P* |
| | -2 | Sleep Duration | 0.005 | 0.868 | 0.008 | 0.802 | 0.008 | 0.800 | 0.038 | 0.230 |
| | | REM Sleep | 0.052 | 0.100 | 0.036 | 0.252 | 0.018 | 0.559 | 0.014 | 0.656 |
| | | Deep Sleep | 0.022 | 0.487 | 0.062 | 0.051 | 0.010 | 0.756 | 0.045 | 0.157 |
| | | HRV (RMSSD) | -0.028 | 0.370 | -0.015 | 0.638 | -0.058 | 0.064 | -0.003 | 0.915 |
| | | HR | **0.107** | **0.001** | **0.063** | **0.046** | **0.079** | **0.012** | -0.029 | 0.367 |
| | | RR | -0.030 | 0.335 | -0.055 | 0.080 | 0.058 | 0.064 | -0.018 | 0.566 |
| | | Temp Deviation | 0.010 | 0.753 | -0.010 | 0.758 | 0.034 | 0.278 | 0.013 | 0.676 |
| | -1 | Sleep Duration | -0.017 | 0.587 | -0.014 | 0.659 | -0.029 | 0.358 | -0.001 | 0.976 |
| | | REM Sleep | 0.019 | 0.551 | -0.007 | 0.835 | -0.013 | 0.670 | -0.041 | 0.202 |
| | | Deep Sleep | 0.041 | 0.188 | 0.040 | 0.208 | 0.052 | 0.095 | 0.022 | 0.481 |
| | | HRV (RMSSD) | 0.005 | 0.878 | 0.040 | 0.212 | 0.024 | 0.438 | **0.073** | **0.022** |
| | | HR | **0.070** | **0.025** | **0.063** | **0.048** | -0.035 | 0.268 | -0.018 | 0.573 |
| | | RR | -0.040 | 0.205 | -0.053 | 0.093 | -0.003 | 0.935 | -0.033 | 0.299 |
| | | Temp Deviation | -0.014 | 0.658 | -0.003 | 0.916 | 0.015 | 0.627 | 0.027 | 0.390 |
| | 0 | Sleep Duration | -0.017 | 0.590 | -0.008 | 0.792 | -0.022 | 0.479 | 0.014 | 0.648 |
| | | REM Sleep | 0.013 | 0.668 | 0.035 | 0.271 | -0.023 | 0.463 | 0.017 | 0.591 |
| | | Deep Sleep | -0.007 | 0.814 | -0.056 | 0.074 | -0.022 | 0.491 | **-0.114** | **0.000** |
| | | HRV (RMSSD) | -0.010 | 0.754 | **-0.117** | **0.000** | 0.006 | 0.849 | **-0.188** | **0.000** |
| | | HR | **0.072** | **0.022** | **0.203** | **0.000** | -0.009 | 0.785 | **0.195** | **0.000** |
| | | RR | -0.026 | 0.403 | 0.058 | 0.065 | 0.061 | 0.052 | **0.183** | **0.000** |
| | | Temp Deviation | -0.004 | 0.899 | **0.221** | **0.000** | 0.014 | 0.650 | **0.228** | **0.000** |
| | 1 | Sleep Duration | -0.014 | 0.647 | 0.061 | 0.057 | -0.008 | 0.796 | **0.109** | **0.001** |
| | | REM Sleep | 0.036 | 0.253 | **0.073** | **0.022** | 0.005 | 0.863 | **0.067** | **0.035** |
| | | Deep Sleep | 0.029 | 0.359 | 0.010 | 0.752 | 0.020 | 0.525 | -0.020 | 0.541 |
| | | HRV (RMSSD) | -0.032 | 0.301 | -0.025 | 0.428 | **-0.076** | **0.015** | -0.059 | 0.064 |
| | | HR | **0.082** | **0.009** | **0.105** | **0.001** | 0.031 | 0.325 | **0.067** | **0.037** |
| | | RR | -0.005 | 0.876 | **0.071** | **0.026** | **0.095** | **0.002** | **0.228** | **0.000** |
| | | Temp Deviation | 0.026 | 0.407 | **0.211** | **0.000** | 0.049 | 0.121 | **0.219** | **0.000** |
| | 2 | Sleep Duration | 0.025 | 0.425 | -0.059 | 0.065 | 0.039 | 0.208 | -0.062 | 0.051 |
| | | REM Sleep | 0.045 | 0.151 | -0.002 | 0.948 | 0.033 | 0.299 | -0.034 | 0.286 |
| | | Deep Sleep | -0.009 | 0.773 | -0.002 | 0.956 | -0.009 | 0.765 | -0.023 | 0.467 |
| | | HRV (RMSSD) | -0.021 | 0.510 | -0.025 | 0.427 | -0.009 | 0.775 | -0.026 | 0.409 |
| | | HR | **0.083** | **0.008** | **0.072** | **0.024** | 0.012 | 0.704 | -0.017 | 0.591 |
| | | RR | -0.038 | 0.228 | -0.001 | 0.980 | 0.039 | 0.215 | **0.095** | **0.003** |
| | | Temp Deviation | 0.025 | 0.430 | 0.058 | 0.070 | 0.031 | 0.321 | 0.061 | 0.056 |

*Note.* See Table 2 and Table 3 notes for variable descriptions and preparations. Analyses are identical to Table 3, however, retain the *n*=51 participants whose RBD antibody responses were returned from LabCorp as ">250 IU/ml" as having values of 250 IU/ml.

**Table S3**. Spearman rank order correlations between RBD antibody responses and device-generated metrics (retaining *n*=51 values of ">250 IU/ml" as 250 IU/ml) on nights before and after vaccine injections, adjusted for the pre-vaccination baseline period.

| Night Relative to Injection | Metric | Injection 1 | | Injection 1 | | | | Injection 2 | | | |
|---|---|---|---|---|---|---|---|---|---|---|---|
| | | J&J | | Moderna | | Pfizer | | Moderna | | Pfizer | |
| | | rho | *P* | rho | *P* | rho | *P* | rho | *P* | rho | *P* |
| -2 | Sleep Duration | -0.095 | 0.343 | -0.012 | 0.825 | 0.053 | 0.172 | 0.062 | 0.257 | 0.035 | 0.366 |
| | REM Sleep | -0.052 | 0.605 | -0.063 | 0.240 | 0.071 | 0.069 | -0.024 | 0.661 | 0.028 | 0.472 |
| | Deep Sleep | -0.137 | 0.173 | -0.004 | 0.944 | 0.010 | 0.787 | 0.093 | 0.088 | 0.027 | 0.491 |
| | HRV (RMSSD) | -0.105 | 0.297 | -0.006 | 0.910 | **-0.102** | **0.009** | 0.006 | 0.907 | 0.024 | 0.542 |
| | HR | -0.111 | 0.268 | 0.013 | 0.807 | **0.105** | **0.007** | -0.036 | 0.505 | -0.060 | 0.123 |
| | RR | -0.076 | 0.450 | -0.045 | 0.398 | **0.083** | **0.032** | -0.046 | 0.403 | 0.024 | 0.539 |
| | Temp Deviation | -0.052 | 0.605 | -0.017 | 0.752 | 0.067 | 0.084 | 0.037 | 0.494 | -0.005 | 0.903 |
| -1 | Sleep Duration | 0.033 | 0.740 | -0.033 | 0.531 | -0.048 | 0.217 | 0.012 | 0.823 | -0.001 | 0.984 |
| | REM Sleep | -0.016 | 0.874 | -0.066 | 0.209 | -0.037 | 0.335 | -0.079 | 0.148 | -0.035 | 0.368 |
| | Deep Sleep | 0.062 | 0.539 | -0.026 | 0.618 | 0.066 | 0.087 | -0.008 | 0.887 | 0.024 | 0.533 |
| | HRV (RMSSD) | -0.077 | 0.441 | 0.054 | 0.311 | 0.015 | 0.702 | 0.069 | 0.207 | 0.046 | 0.238 |
| | HR | 0.128 | 0.199 | -0.033 | 0.531 | -0.032 | 0.414 | -0.093 | 0.087 | 0.027 | 0.490 |
| | RR | 0.007 | 0.947 | -0.039 | 0.464 | 0.006 | 0.886 | -0.025 | 0.644 | 0.017 | 0.665 |
| | Temp Deviation | 0.004 | 0.971 | 0.004 | 0.945 | -0.012 | 0.758 | 0.076 | 0.163 | -0.004 | 0.910 |
| 0 | Sleep Duration | 0.166 | 0.098 | 0.094 | 0.083 | -0.057 | 0.138 | 0.011 | 0.841 | 0.011 | 0.784 |
| | REM Sleep | 0.106 | 0.293 | 0.025 | 0.641 | -0.035 | 0.367 | 0.038 | 0.488 | 0.005 | 0.899 |
| | Deep Sleep | -0.173 | 0.083 | -0.036 | 0.510 | -0.011 | 0.779 | -0.072 | 0.181 | **-0.100** | **0.011** |
| | HRV (RMSSD) | -0.001 | 0.993 | -0.014 | 0.791 | 0.020 | 0.605 | **-0.158** | **0.003** | **-0.155** | **0.000** |
| | HR | 0.125 | 0.212 | -0.027 | 0.620 | -0.042 | 0.276 | **0.158** | **0.003** | **0.111** | **0.004** |
| | RR | 0.113 | 0.262 | 0.035 | 0.516 | 0.054 | 0.160 | **0.181** | **0.001** | **0.112** | **0.004** |
| | Temp Deviation | 0.141 | 0.160 | 0.026 | 0.624 | -0.013 | 0.744 | **0.157** | **0.003** | **0.139** | **0.000** |
| 1 | Sleep Duration | **-0.224** | **0.025** | -0.062 | 0.248 | 0.034 | 0.382 | 0.045 | 0.410 | **0.082** | **0.038** |
| | REM Sleep | **-0.281** | **0.005** | 0.043 | 0.423 | -0.010 | 0.798 | 0.066 | 0.228 | 0.059 | 0.138 |
| | Deep Sleep | -0.152 | 0.130 | -0.026 | 0.630 | 0.054 | 0.164 | -0.102 | 0.063 | 0.003 | 0.936 |
| | HRV (RMSSD) | 0.002 | 0.986 | -0.046 | 0.392 | -0.057 | 0.146 | -0.009 | 0.863 | -0.040 | 0.309 |
| | HR | 0.042 | 0.677 | -0.031 | 0.565 | -0.009 | 0.810 | 0.018 | 0.737 | 0.046 | 0.243 |
| | RR | 0.057 | 0.573 | **0.108** | **0.043** | 0.041 | 0.290 | **0.126** | **0.022** | **0.233** | **0.000** |
| | Temp Deviation | 0.067 | 0.511 | 0.085 | 0.113 | -0.024 | 0.535 | **0.127** | **0.021** | **0.169** | **0.000** |
| 2 | Sleep Duration | 0.030 | 0.769 | -0.004 | 0.938 | 0.047 | 0.223 | -0.037 | 0.505 | -0.048 | 0.219 |
| | REM Sleep | -0.125 | 0.214 | -0.019 | 0.728 | 0.022 | 0.575 | -0.049 | 0.377 | -0.022 | 0.569 |
| | Deep Sleep | 0.052 | 0.602 | -0.004 | 0.940 | -0.008 | 0.841 | 0.043 | 0.437 | -0.054 | 0.173 |
| | HRV (RMSSD) | -0.039 | 0.701 | 0.039 | 0.467 | -0.026 | 0.498 | 0.018 | 0.738 | **-0.079** | **0.043** |
| | HR | 0.111 | 0.269 | -0.014 | 0.797 | 0.014 | 0.715 | -0.034 | 0.542 | 0.013 | 0.749 |
| | RR | 0.082 | 0.416 | 0.049 | 0.358 | 0.026 | 0.510 | 0.082 | 0.132 | **0.088** | **0.024** |
| | Temp Deviation | 0.195 | 0.051 | 0.043 | 0.420 | -0.006 | 0.878 | **0.117** | **0.032** | -0.006 | 0.874 |

*Note.* See Table 2 and Table 3 notes for variable descriptions and preparations. Analyses are identical to Table 4, however, retain the *n*=51 participants whose RBD antibody responses were returned from LabCorp as ">250 IU/ml" (rather than a specific value).

**Table S4.** Kendall rank order correlations between RBD antibody responses and device-generated metrics on nights before and after vaccine injections.

| Night Relative to Injection | | Metric | Injection 1 | | Injection 1 | | | | Injection 2 | | | |
|---|---|---|---|---|---|---|---|---|---|---|---|---|
| | | | J&J | | Moderna | | Pfizer | | Moderna | | Pfizer | |
| | | | τ | *P* | τ | *P* | τ | *P* | τ | *P* | τ | *P* |
| | -2 | Sleep Duration | -0.086 | 0.204 | -0.024 | 0.574 | 0.011 | 0.692 | 0.053 | 0.219 | -0.016 | 0.563 |
| | | REM Sleep | 0.019 | 0.774 | -0.029 | 0.494 | **0.073** | **0.008** | 0.007 | 0.866 | 0.043 | 0.124 |
| | | Deep Sleep | -0.095 | 0.162 | -0.019 | 0.647 | 0.006 | 0.829 | 0.054 | 0.213 | 0.034 | 0.222 |
| | | HRV (RMSSD) | -0.076 | 0.262 | 0.012 | 0.782 | -0.033 | 0.237 | 0.006 | 0.885 | 0.017 | 0.543 |
| | | HR | -0.022 | 0.747 | 0.025 | 0.553 | **0.096** | **0.000** | 0.025 | 0.561 | 0.043 | 0.120 |
| | | RR | 0.053 | 0.436 | -0.038 | 0.372 | -0.048 | 0.082 | **-0.102** | **0.019** | -0.033 | 0.243 |
| | | Temp Deviation | -0.009 | 0.890 | -0.009 | 0.833 | 0.037 | 0.186 | 0.033 | 0.446 | -0.004 | 0.884 |
| | -1 | Sleep Duration | 0.025 | 0.716 | -0.017 | 0.685 | -0.031 | 0.261 | -0.043 | 0.314 | -0.048 | 0.086 |
| | | REM Sleep | 0.018 | 0.792 | -0.048 | 0.253 | 0.022 | 0.418 | -0.064 | 0.134 | 0.001 | 0.971 |
| | | Deep Sleep | 0.005 | 0.942 | -0.036 | 0.392 | 0.032 | 0.242 | 0.005 | 0.904 | 0.033 | 0.241 |
| | | HRV (RMSSD) | -0.054 | 0.425 | 0.038 | 0.363 | 0.000 | 0.996 | 0.021 | 0.627 | 0.031 | 0.267 |
| | | HR | 0.036 | 0.589 | 0.015 | 0.716 | **0.072** | **0.009** | 0.014 | 0.748 | **0.064** | **0.021** |
| | | RR | 0.085 | 0.211 | -0.036 | 0.394 | **-0.067** | **0.016** | -0.037 | 0.392 | **-0.056** | **0.044** |
| | | Temp Deviation | -0.028 | 0.683 | -0.017 | 0.688 | 0.002 | 0.955 | 0.041 | 0.344 | -0.016 | 0.566 |
| | 0 | Sleep Duration | 0.084 | 0.213 | 0.049 | 0.246 | **-0.056** | **0.041** | -0.003 | 0.940 | -0.024 | 0.396 |
| | | REM Sleep | 0.033 | 0.628 | 0.036 | 0.399 | -0.001 | 0.961 | 0.019 | 0.652 | 0.024 | 0.395 |
| | | Deep Sleep | -0.111 | 0.100 | -0.007 | 0.861 | 0.003 | 0.908 | -0.019 | 0.654 | -0.040 | 0.147 |
| | | HRV (RMSSD) | -0.029 | 0.668 | 0.008 | 0.850 | 0.005 | 0.870 | -0.044 | 0.308 | **-0.064** | **0.023** |
| | | HR | 0.085 | 0.209 | 0.006 | 0.889 | **0.070** | **0.011** | **0.105** | **0.014** | **0.123** | **0.000** |
| | | RR | 0.085 | 0.214 | -0.039 | 0.362 | -0.041 | 0.141 | -0.016 | 0.707 | -0.004 | 0.892 |
| | | Temp Deviation | 0.112 | 0.097 | -0.008 | 0.857 | -0.002 | 0.956 | **0.096** | **0.024** | **0.107** | **0.000** |
| | 1 | Sleep Duration | **-0.135** | **0.047** | -0.041 | 0.330 | -0.001 | 0.961 | 0.053 | 0.223 | -0.005 | 0.864 |
| | | REM Sleep | -0.035 | 0.606 | 0.016 | 0.699 | 0.030 | 0.278 | 0.054 | 0.215 | **0.057** | **0.040** |
| | | Deep Sleep | -0.089 | 0.189 | -0.004 | 0.919 | 0.029 | 0.298 | -0.068 | 0.120 | 0.018 | 0.516 |
| | | HRV (RMSSD) | -0.045 | 0.508 | 0.001 | 0.981 | -0.009 | 0.754 | 0.009 | 0.835 | -0.011 | 0.690 |
| | | HR | 0.036 | 0.598 | 0.001 | 0.976 | **0.061** | **0.028** | 0.040 | 0.359 | **0.080** | **0.004** |
| | | RR | 0.111 | 0.107 | -0.024 | 0.576 | -0.046 | 0.097 | -0.005 | 0.909 | 0.034 | 0.228 |
| | | Temp Deviation | 0.066 | 0.334 | 0.042 | 0.316 | -0.021 | 0.446 | **0.104** | **0.017** | **0.129** | **0.000** |
| | 2 | Sleep Duration | 0.032 | 0.639 | -0.016 | 0.695 | -0.012 | 0.660 | -0.047 | 0.280 | **-0.075** | **0.007** |
| | | REM Sleep | 0.010 | 0.883 | -0.020 | 0.635 | 0.021 | 0.438 | -0.029 | 0.508 | -0.001 | 0.977 |
| | | Deep Sleep | 0.022 | 0.740 | -0.029 | 0.487 | -0.005 | 0.867 | 0.008 | 0.845 | -0.010 | 0.715 |
| | | HRV (RMSSD) | -0.042 | 0.538 | 0.029 | 0.498 | 0.004 | 0.896 | 0.005 | 0.913 | -0.029 | 0.307 |
| | | HR | 0.078 | 0.248 | 0.025 | 0.549 | **0.062** | **0.025** | 0.031 | 0.481 | **0.069** | **0.014** |
| | | RR | 0.098 | 0.153 | -0.001 | 0.981 | **-0.065** | **0.020** | -0.038 | 0.385 | -0.021 | 0.453 |
| | | Temp Deviation | 0.130 | 0.055 | 0.000 | 0.998 | -0.013 | 0.628 | 0.062 | 0.152 | 0.014 | 0.617 |

*Note*. See Table 2 note. Kendall rank order correlations directly account for tied ranks and better manage Type 1 error rates (see Materials and Methods).

**Table S5.** Kendall rank order correlations between RBD antibody responses and device-generated metrics before and after adjusting by the pre-vaccination baseline period on nights before and after injections for Moderna-NIAID and Pfizer-BioNTech vaccine recipients, combined.

| Night Relative to Injection | Metric | Device-generated Metric | | | | Adjusted for Baseline period | | | |
|---|---|---|---|---|---|---|---|---|---|
| | | Injection 1 | | Injection 2 | | Injection 1 | | Injection 2 | |
| | | τ | *P* | τ | *P* | τ | *P* | τ | *P* |
| -2 | Sleep Duration | -0.001 | 0.949 | 0.015 | 0.526 | 0.000 | 0.995 | 0.044 | 0.059 |
| | REM Sleep | 0.041 | 0.074 | 0.042 | 0.070 | 0.020 | 0.388 | 0.027 | 0.246 |
| | Deep Sleep | -0.006 | 0.788 | 0.030 | 0.196 | -0.003 | 0.882 | 0.029 | 0.211 |
| | HRV (RMSSD) | -0.022 | 0.334 | -0.005 | 0.818 | **-0.047** | **0.041** | 0.002 | 0.916 |
| | HR | **0.072** | **0.002** | 0.036 | 0.119 | **0.058** | **0.012** | -0.016 | 0.489 |
| | RR | -0.031 | 0.183 | **-0.052** | **0.027** | 0.041 | 0.071 | -0.008 | 0.728 |
| | Temp Deviation | 0.012 | 0.603 | 0.001 | 0.973 | 0.031 | 0.172 | 0.016 | 0.495 |
| -1 | Sleep Duration | -0.008 | 0.724 | -0.030 | 0.201 | -0.007 | 0.750 | -0.016 | 0.482 |
| | REM Sleep | 0.026 | 0.266 | -0.011 | 0.644 | 0.005 | 0.815 | -0.036 | 0.121 |
| | Deep Sleep | 0.012 | 0.596 | 0.022 | 0.343 | 0.040 | 0.080 | 0.017 | 0.472 |
| | HRV (RMSSD) | -0.001 | 0.972 | 0.031 | 0.188 | 0.008 | 0.719 | **0.052** | **0.025** |
| | HR | **0.050** | **0.030** | 0.037 | 0.114 | -0.006 | 0.783 | -0.008 | 0.740 |
| | RR | -0.041 | 0.074 | **-0.047** | **0.046** | 0.006 | 0.781 | -0.018 | 0.439 |
| | Temp Deviation | 0.005 | 0.815 | -0.011 | 0.652 | 0.026 | 0.262 | 0.013 | 0.591 |
| 0 | Sleep Duration | -0.017 | 0.471 | -0.009 | 0.692 | -0.013 | 0.573 | 0.017 | 0.461 |
| | REM Sleep | 0.007 | 0.762 | 0.023 | 0.332 | -0.017 | 0.466 | 0.016 | 0.497 |
| | Deep Sleep | -0.019 | 0.420 | **-0.057** | **0.014** | -0.010 | 0.656 | **-0.091** | **0.000** |
| | HRV (RMSSD) | -0.007 | 0.751 | **-0.085** | **0.000** | -0.005 | 0.826 | **-0.143** | **0.000** |
| | HR | **0.050** | **0.030** | **0.141** | **0.000** | 0.008 | 0.717 | **0.149** | **0.000** |
| | RR | -0.029 | 0.219 | 0.027 | 0.251 | **0.050** | **0.028** | **0.127** | **0.000** |
| | Temp Deviation | 0.005 | 0.843 | **0.172** | **0.000** | 0.017 | 0.472 | **0.176** | **0.000** |
| 1 | Sleep Duration | -0.013 | 0.562 | **0.047** | **0.043** | -0.011 | 0.638 | **0.090** | **0.000** |
| | REM Sleep | 0.023 | 0.312 | **0.058** | **0.013** | 0.007 | 0.749 | **0.060** | **0.010** |
| | Deep Sleep | 0.010 | 0.659 | -0.009 | 0.711 | 0.020 | 0.377 | -0.014 | 0.548 |
| | HRV (RMSSD) | -0.023 | 0.312 | -0.020 | 0.388 | **-0.057** | **0.013** | **-0.054** | **0.022** |
| | HR | **0.052** | **0.025** | **0.071** | **0.002** | 0.024 | 0.286 | **0.059** | **0.012** |
| | RR | -0.011 | 0.638 | **0.048** | **0.042** | **0.070** | **0.002** | **0.187** | **0.000** |
| | Temp Deviation | 0.029 | 0.212 | **0.174** | **0.000** | **0.046** | **0.044** | **0.180** | **0.000** |
| 2 | Sleep Duration | 0.007 | 0.766 | **-0.061** | **0.010** | 0.025 | 0.276 | **-0.060** | **0.010** |
| | REM Sleep | 0.026 | 0.260 | -0.009 | 0.695 | 0.022 | 0.334 | -0.034 | 0.143 |
| | Deep Sleep | -0.017 | 0.457 | -0.015 | 0.530 | -0.013 | 0.586 | -0.022 | 0.339 |
| | HRV (RMSSD) | -0.001 | 0.974 | -0.023 | 0.332 | 0.005 | 0.812 | -0.036 | 0.124 |
| | HR | **0.048** | **0.035** | **0.048** | **0.040** | 0.017 | 0.470 | 0.004 | 0.873 |
| | RR | -0.038 | 0.102 | -0.007 | 0.775 | 0.040 | 0.083 | **0.083** | **0.000** |
| | Temp Deviation | 0.009 | 0.708 | **0.048** | **0.042** | 0.015 | 0.504 | **0.054** | **0.022** |

*Note.* See Table 2 and Table 3 notes for variable descriptions and preparations. See also Table S4 note. Physiological metric pre-vaccination baseline period taken from nights -14 to -4 prior to first injection.

**Table S6.** Kendall rank order correlations between RBD antibody responses and device-generated metrics on nights before and after vaccine injections, adjusted for the pre-vaccination baseline period.

| Night Relative to Injection | Metric | Injection 1 | | Injection 1 | | | | Injection 2 | | | |
|---|---|---|---|---|---|---|---|---|---|---|---|
| | | J&J | | Moderna | | Pfizer | | Moderna | | Pfizer | |
| | | τ | *P* | τ | *P* | τ | *P* | τ | *P* | τ | *P* |
| -2 | Sleep Duration | -0.061 | 0.366 | -0.025 | 0.557 | 0.036 | 0.185 | **0.098** | **0.023** | 0.032 | 0.251 |
| | REM Sleep | -0.037 | 0.585 | -0.040 | 0.344 | **0.056** | **0.042** | 0.011 | 0.794 | 0.032 | 0.246 |
| | Deep Sleep | -0.103 | 0.127 | -0.009 | 0.836 | -0.006 | 0.841 | 0.056 | 0.193 | 0.020 | 0.461 |
| | HRV (RMSSD) | -0.072 | 0.285 | -0.027 | 0.522 | **-0.071** | **0.010** | 0.038 | 0.379 | 0.015 | 0.593 |
| | HR | -0.079 | 0.243 | 0.022 | 0.608 | **0.070** | **0.011** | -0.021 | 0.633 | -0.041 | 0.142 |
| | RR | -0.052 | 0.446 | -0.004 | 0.929 | 0.045 | 0.103 | -0.036 | 0.410 | 0.025 | 0.362 |
| | Temp Deviation | -0.039 | 0.561 | 0.029 | 0.486 | 0.041 | 0.140 | 0.056 | 0.191 | -0.004 | 0.898 |
| -1 | Sleep Duration | 0.032 | 0.637 | -0.006 | 0.877 | -0.024 | 0.385 | -0.012 | 0.783 | -0.014 | 0.611 |
| | REM Sleep | -0.008 | 0.901 | -0.049 | 0.241 | -0.009 | 0.734 | -0.082 | 0.056 | -0.028 | 0.314 |
| | Deep Sleep | 0.044 | 0.512 | -0.022 | 0.590 | 0.048 | 0.082 | 0.011 | 0.805 | 0.010 | 0.706 |
| | HRV (RMSSD) | -0.051 | 0.447 | 0.027 | 0.510 | 0.004 | 0.883 | 0.040 | 0.356 | 0.034 | 0.224 |
| | HR | 0.089 | 0.184 | 0.008 | 0.839 | -0.007 | 0.787 | -0.040 | 0.352 | 0.016 | 0.565 |
| | RR | -0.001 | 0.993 | -0.006 | 0.889 | 0.006 | 0.830 | 0.044 | 0.308 | 0.003 | 0.913 |
| | Temp Deviation | 0.003 | 0.961 | 0.025 | 0.556 | 0.002 | 0.950 | 0.054 | 0.211 | -0.012 | 0.675 |
| 0 | Sleep Duration | 0.108 | 0.110 | 0.073 | 0.088 | -0.033 | 0.228 | 0.012 | 0.784 | 0.016 | 0.574 |
| | REM Sleep | 0.068 | 0.313 | 0.042 | 0.325 | -0.030 | 0.271 | 0.028 | 0.511 | 0.009 | 0.753 |
| | Deep Sleep | -0.119 | 0.077 | -0.007 | 0.862 | -0.007 | 0.802 | -0.042 | 0.320 | **-0.084** | **0.002** |
| | HRV (RMSSD) | -0.003 | 0.963 | -0.034 | 0.419 | 0.010 | 0.728 | **-0.091** | **0.031** | **-0.126** | **0.000** |
| | HR | 0.092 | 0.175 | -0.020 | 0.637 | -0.014 | 0.606 | **0.114** | **0.007** | **0.086** | **0.002** |
| | RR | 0.077 | 0.252 | 0.006 | 0.886 | 0.052 | 0.059 | **0.108** | **0.011** | **0.079** | **0.004** |
| | Temp Deviation | 0.113 | 0.096 | 0.016 | 0.710 | -0.001 | 0.983 | **0.122** | **0.004** | **0.107** | **0.000** |
| 1 | Sleep Duration | **-0.150** | **0.027** | -0.053 | 0.208 | 0.018 | 0.512 | 0.068 | 0.116 | **0.056** | **0.044** |
| | REM Sleep | **-0.186** | **0.006** | 0.044 | 0.297 | -0.006 | 0.818 | 0.084 | 0.052 | 0.046 | 0.103 |
| | Deep Sleep | -0.115 | 0.090 | 0.019 | 0.652 | 0.032 | 0.240 | **-0.092** | **0.035** | 0.004 | 0.873 |
| | HRV (RMSSD) | 0.001 | 0.990 | -0.031 | 0.454 | -0.041 | 0.132 | -0.004 | 0.926 | -0.042 | 0.137 |
| | HR | 0.027 | 0.692 | -0.038 | 0.368 | -0.004 | 0.874 | 0.032 | 0.454 | 0.037 | 0.183 |
| | RR | 0.043 | 0.522 | 0.079 | 0.060 | 0.030 | 0.283 | **0.127** | **0.003** | **0.180** | **0.000** |
| | Temp Deviation | 0.055 | 0.416 | 0.072 | 0.087 | -0.007 | 0.796 | **0.128** | **0.003** | **0.127** | **0.000** |
| 2 | Sleep Duration | 0.022 | 0.747 | -0.004 | 0.923 | 0.027 | 0.322 | -0.050 | 0.251 | -0.046 | 0.102 |
| | REM Sleep | -0.080 | 0.234 | -0.015 | 0.722 | 0.011 | 0.698 | -0.063 | 0.144 | -0.021 | 0.453 |
| | Deep Sleep | 0.025 | 0.716 | -0.014 | 0.740 | -0.009 | 0.734 | 0.035 | 0.412 | -0.046 | 0.100 |
| | HRV (RMSSD) | -0.026 | 0.699 | 0.059 | 0.159 | -0.011 | 0.687 | 0.004 | 0.921 | **-0.076** | **0.006** |
| | HR | 0.074 | 0.272 | 0.008 | 0.855 | 0.014 | 0.614 | 0.001 | 0.981 | 0.023 | 0.406 |
| | RR | 0.054 | 0.420 | 0.076 | 0.069 | 0.021 | 0.446 | **0.107** | **0.014** | **0.066** | **0.018** |
| | Temp Deviation | **0.137** | **0.043** | 0.034 | 0.416 | -0.016 | 0.556 | **0.096** | **0.027** | 0.005 | 0.848 |

*Note.* See Table 2 and Table 3 notes for variable descriptions and preparations. See also Table S4 note.
